# Conidiation Color Mutants of *Aspergillus fumigatus* Are Highly Pathogenic to the Heterologous Insect Host *Galleria mellonella*

**DOI:** 10.1371/journal.pone.0004224

**Published:** 2009-01-19

**Authors:** Jennifer C. Jackson, Laura A. Higgins, Xiaorong Lin

**Affiliations:** Department of Biology, Texas A & M University, College Station, Texas, United States of America; Massachusetts General Hospital, United States of America

## Abstract

The greater wax moth *Galleria mellonella* has been widely used as a heterologous host for a number of fungal pathogens including *Candida albicans* and *Cryptococcus neoformans*. A positive correlation in pathogenicity of these yeasts in this insect model and animal models has been observed. However, very few studies have evaluated the possibility of applying this heterologous insect model to investigate virulence traits of the filamentous fungal pathogen *Aspergillus fumigatus*, the leading cause of invasive aspergillosis. Here, we have examined the impact of mutations in genes involved in melanin biosynthesis on the pathogenicity of *A. fumigatus* in the *G. mellonella* model. Melanization in *A. fumigatus* confers bluish-grey color to conidia and is a known virulence factor in mammal models. Surprisingly, conidial color mutants in B5233 background that have deletions in the defined six-gene cluster required for DHN-melanin biosynthesis caused enhanced insect mortality compared to the parent strain. To further examine and confirm the relationship between melanization defects and enhanced virulence in the wax moth model, we performed random insertional mutagenesis in the Af293 genetic background to isolate mutants producing altered conidia colors. Strains producing conidia of previously identified colors and of novel colors were isolated. Interestingly, these color mutants displayed a higher level of pathogenicity in the insect model compared to the wild type. Although some of the more virulent color mutants showed increased resistance to hydrogen peroxide, overall phenotypic characterizations including secondary metabolite production, metalloproteinase activity, and germination rate did not reveal a general mechanism accountable for the enhanced virulence of these color mutants observed in the insect model. Our observations indicate instead, that exacerbated immune response of the wax moth induced by increased exposure of PAMPs (pathogen-associated molecular patterns) may cause self-damage that results in increased mortality of larvae infected with the color mutants. The current study underscores the limitations of using this insect model for inferring the pathogenic potential of *A. fumigatus* strains in mammals, but also points to the importance of understanding the innate immunity of the insect host in providing insights into the pathogenicity level of different fungal strains in this model. Additionally, our observations that melanization defective color mutants demonstrate increased virulence in the insect wax moth, suggest the potential of using melanization defective mutants of native insect fungal pathogens in the biological control of insect populations.

## Introduction

Invertebrates have been increasingly viewed as a valid model for studying the virulence of microbial human pathogens because of the similarities in the basic innate immune systems between these non-vertebrate hosts and mammals [1]–[3]. Furthermore, the virulence mechanisms of microbial pathogens, especially of environmental opportunistic pathogens, are likely conserved among different hosts as these pathogenic microbes often have evolved and maintained their virulence through interactions with a diverse range of environmental predators like amoeba or nematodes [4]–[7].

Popular non-vertebrate hosts for virulence studies of human pathogenic fungi include the nematode *Caenorhabditis elegans* [8]–[11], the greater wax moth *Galleria mellonella* [12]–[16], and the fruit fly *Drosophila melanogaster* [17]–[20]. The caterpillar *G. mellonella* is an attractive insect host for testing fungal virulence and antifungal agents due to its easy handling, low cost, and low maintenance [12], [14], [21]. In addition, its antifungal immunity can be examined by a variety of assays [1], [22]–[24]. Although the insect cuticle is the first line of defense against the majority of pathogens in nature, exposure to the cuticle is often not adopted as the primary infection route in the laboratory due to the fact that most human fungal pathogens are not natural pathogens of *G. mellonella*. Instead, injection of fungal cells through the insect prolegs is routinely used [12]–[16], [25]. The insect amounts both cellular and humoral immune responses upon recognizing microbes in the body cavity [1], [26], [27]. The cellular defenses rely on the response of the insect immune cells (haemocytes) and activated enzyme cascades such as phenoloxidase cascade. The haemocytes in insect haemolymph (similar to mammal blood) activate phagocytosis, nodule formation, and encapsulation to clear and/or restrict fungal cells. Nodules can be rapidly melanized through the prophenoloxidase pathway during cellular defense reactions. Haemolymph clotting and production of anti-microbial peptides and proteases are the typical insect humoral responses against pathogens.

A positive correlation in pathogenicity for this insect model and animal models has been demonstrated for several fungal pathogens including *Cryptococcus neoformans* and *Candida albicans*. Virulence factors that are important for *C. neoformans* and *C. albicans* to infect mammalian animals are also shown to be necessary for the pathogenicity in the caterpillar [1], [10], [13], [15], [16], [23]. At present, *G. mellonella* is routinely used as one of the non-vertebrate hosts for testing virulence traits in these organisms.

The common mold *A. fumigatus* is the leading cause of various aspergillosis diseases including invasive aspergillosis in individuals with leukemia, tuberculosis, or other cystic lung diseases [28], [29]. It also infects recipients of bone marrow and solid organ transplantations [30], [31]. Fatality rates greater than 50% are common among patients with invasive aspergillosis even with aggressive antifungal therapies [29], [32], [33]. Because of the poor outcome of current treatments, it is imperative to understand the nature of *A. fumigatus* pathogenesis and to develop more effective therapies for combating invasive aspergillosis in humans. Given the importance of studying the nature of *A. fumigatus* pathogenesis, it becomes necessary to explore potential alternative host models for *A. fumigatus* and to know the limitations therein. Properly using these alternative hosts to study *A. fumigatus* virulence traits that are relevant in mammalian host could greatly facilitate the advancement of our understanding of its virulence strategies and the development of more efficient treatments for aspergillosis.

A previous study found a positive correlation between the level of gliotoxin production in several *A. fumigatus* clinical isolates and their pathogenicity level in greater wax moths [12]. The study suggests that the *G. mellonella* infection model could potentially be applied to *A. fumigatus* pathogenesis studies. Here, we chose to examine the impact of mutations in *A. fumigatus* melanization pathway on its pathogenesis in the *G. mellonella* model. Melanin is a class of polymers formed by oxidative polymerization of phenolic or indolic compounds that confer pigmentations (brown, black etc.). It is found in protists, plants, fungi, and animals, and has a variety of biological functions [34]. For example, melanin in fungi serves as a protectant from harmful UV and solar radiation, it also neutralizes free radicals and provides structural rigidity to cell walls [35]–[39]. Two types of melanin have been well characterized in fungi: the dihydroxyphenylalanine (DOPA)-melanin and dihydroxynaphthalene (DHN)-melanin. In human pathogenic fungi, DOPA-melanin is best characterized in *C. neoformans*. It is one of the major defined virulence factors that enable this organism to infect both mammals and insects including *G. mellonella* [10], [13], [40]–[42]. DHN-melanin has also been implicated in the virulence of *Wangiella dermatitidis* and *A. fumigatus* [43]–[46].

## Results

### 1. Determining the appropriate inoculum of *A. fumigatus* conidia for the *G. mellonella* model

Previously, several *A. fumigatus* clinical strains were shown to be avirulent to *G. mellonella*. The larvae were infected with an inoculum concentration of 3000 conidia per larva and infected larvae were incubated at room temperature [47]. A more recent study showed that conidia of *A. fumigatus* strain ATCC 26933 were avirulent to *G. mellonella* grown at 30°C when inocula less than 1×10^7^ conidia/caterpillar were used [21].

**Figure 1:**
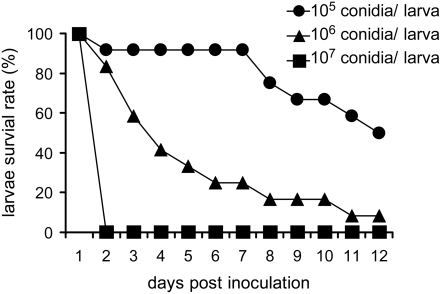
Dose dependent killing of *G. mellonella* larvae by *A. fumigatus*. *G. mellonella* larvae were inoculated with conidia of *A. fumigatus* strains Af293 at 1×10^5^, 1×10^6^, and 1×10^7^ conidia per larva. The rate of *G. mellonella* survival was plotted against days post inoculation.

**Figure 2:**
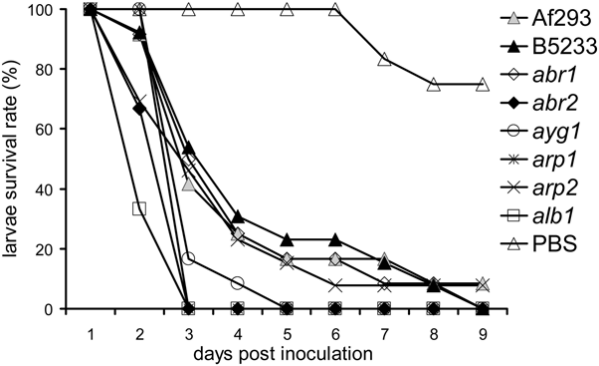
*A. fumigatus* defined color mutants in the B5233 background show enhanced virulence in the *G. mellonella* model. Larvae were inoculated with *A. fumigatus* at concentrations of 1×10^6^ conidia per caterpillar. The *G. mellonella* survival versus days post inoculation was plotted. Wild type strains Af293 and B5233 have similar virulence (p = 0.9601), while color mutants *abr2* (p = 0.0017), *ayg1* (p = 0.0009), *arp1* (p = 0.0050), *arp2* (p = 0.0407), and *alb1* (p<0.0001) have increased virulence compared to the wild type strain. Only the color mutant *abr1* (p = 0.1841) does not have a statistically significant difference in virulence from the wild type strain. Twelve caterpillars were inoculated per *A. fumigatus* strain and studies were repeated three times for each strain and similar pattern was observed.

Because the sequenced clinical strain Af293 is widely used by the *A. fumigatus* research community, we decided to test the pathogenicity level of this strain to *G. mellonella* larvae and to determine the optimal conidial dosage for virulence studies in this insect model. Each larva was injected with 1×10^5^, 1×10^6^, and 1×10^7^ conidia and was incubated at 37°C, the human body temperature. The survival of the infected larvae was monitored daily and was measured as a function of time. As shown in Figure 1, at the inoculation density of 1×10^5^ conidia per insect, strain Af293 was not virulent to *G. mellonella*. However, the fungus killed all of the wax moth larvae within 24 hours when the inoculum of 1×10^7^ conidia/insect was used. At the dose of 1×10^6^ conidia/insect, the fungus showed an intermediate pathogenicity level (Figure 1). Since the dose of 1×10^6^ conidia/larva should allow us to discern discrete differences in virulence potential, this concentration was chosen as the optimal dosage for the subsequent virulence studies described in this paper. A concentration of 1×10^6^ conidia/insect could also be an appropriate dose for other *A. fumigatus* isolates, as another clinical strain, B5233, which is frequently used in genetic and pathogenic studies and is genetically distinct from Af293, also yielded a similar virulence profile as the Af293 strain under the same conditions (Figure 2).

### 2. Mutations in the melanin biosynthesis six-gene cluster in the strain B5233 background caused enhanced virulence to *G. mellonella*

**Figure 3:**
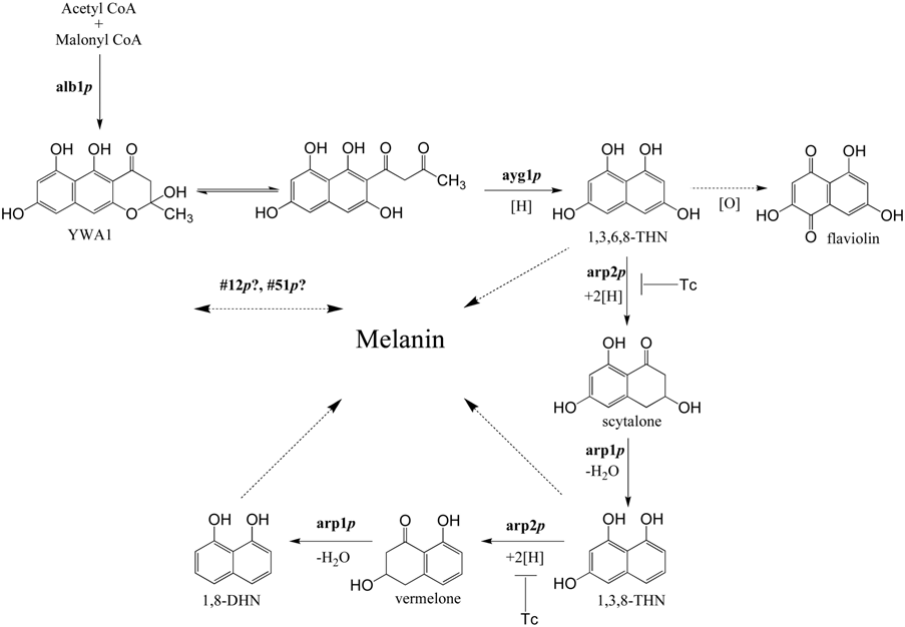
Schematic representation of the biosynthesis of DHN-melanin in *A. fumigatus*. Solid arrows indicate the previously defined melanin pathway. Dashed arrows indicate possible branching pathways from the main melanin pathway. Reduction [H], oxidation [O], and dehydration [−H_2_O] steps are indicated accordingly. Tc indicates a reduction step that can be inhibited by the fungicide tricyclazole.

*A. fumigatus* wild type conidia are bluish-grey in color due to the accumulation of DHN-melanin. The biosynthesis of DHN-melanin in *A. fumigatus* begins with the conversion of acetate to 1,3,6,8-tetrahydroxynaphthalene (1,3,6,8-THN) by polyketide synthase, followed by reduction to scytalone by hydroxynaphthalene (HN) reductase. Scytalone dehydratase catalyzes the dehydration of scytalone to 1,3,8-trihydroxynaphthalene (1,3,8-THN), which can be converted to 1,8-DHN following further reduction and dehydration steps by HN reductase and a dehydratase. Oxidative polymerization of 1,8-DHN give rise to the DHN-melanin [48], [49] (Figure 3).

The six gene cluster (*alb1*, *arp2*, *arp1*, *abr1*, *abr2*, and *ayg1*) that is required for *A. fumigatus* melanization has been characterized previously [49]–[53] (Figure 3). In order to evaluate the role of *A. fumigatus* melanin on the pathogenesis in wax moth, strains containing the targeted deletion of the six genes encoding polyketide synthase (*alb1*), heptaketide hydrolyase (*ayg1*), hydroxynaphthalene reductases (*arp2*), scytalone dehydratases (*arp1*), multicopper oxidase (*abr1*), and laccase (*abr2*) in the strain B5233 background were examined for their virulence in this host. As shown in Figure 2, *alb1*, *abr2*, and *arp1* were highly virulent to the insect and killed all the larvae within 3 days post inoculation. The mutant *ayg1* was modestly more virulent than the wild type strain. Other color mutants such as *arp2* and *abr1* were at least as virulent as the wild type strain B5233. This experiment was repeated three times and similar patterns were observed (data not shown).

**Figure 4:**
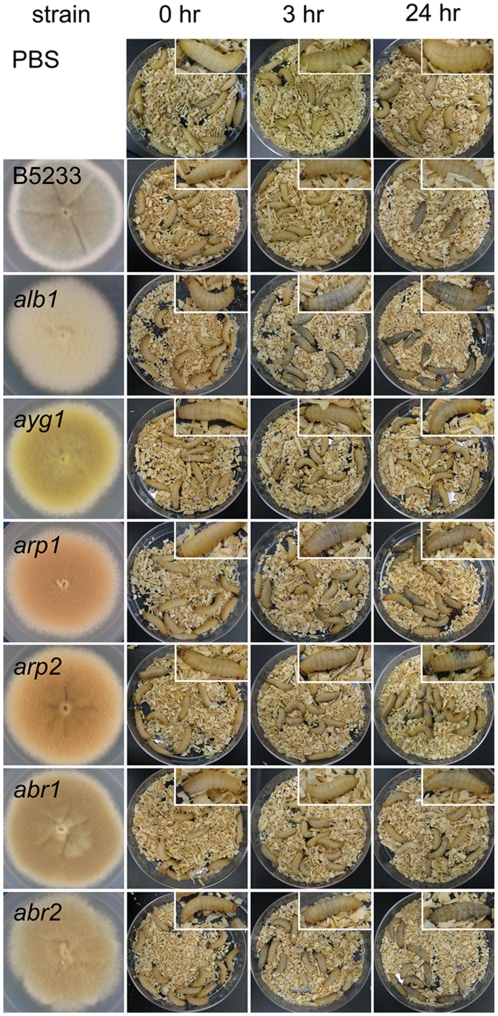
Infection with *A. fumigatus* color mutants in the B5233 background causes *G. mellonella* larvae to darken shortly after inoculation. Larvae were infected with *A. fumigatus* B5233 wild type strain and color mutants *alb1*, *ayg1*, *arp1*, *arp2*, *abr1*, and *abr2* at the concentration of 1×10^6^ conidia per caterpillar. From left to right are images of *A. fumigatus* colonies (columns 1), *G. mellonella* larvae at T = 0 h (columns 2), 3 h (columns 3), and 24 h (columns 4) post infection. Insets are close-up images of a representative single caterpillar from the same plate. Larvae injected with PBS buffer are shown on the top for comparison.

We readily observed that larvae infected with the color mutant spores darkened shortly after inoculation. Typically, larva darkening becomes apparent by 3 hours post inoculation (Figure 4) although these darkened larvae do not die until approximately 2 days later. In contrast, larvae injected with PBS or wild type strain B5233 remain pale in color even at 24 hours post inoculation (Figure 4 and data not shown). There appears to be a positive correlation between the degree of larvae darkening and the pathogenicity level of the strain to the larvae. It is known that infected *G. mellonella* larvae form melanotic capsules surrounding pathogens [1], [26], and the observed darkening of the infected larvae is likely due to the formation of these melanotic capsules.

### 3. Isolation of conidiation color mutants in the *A. fumigatus* strain Af293 background

**Figure 5:**
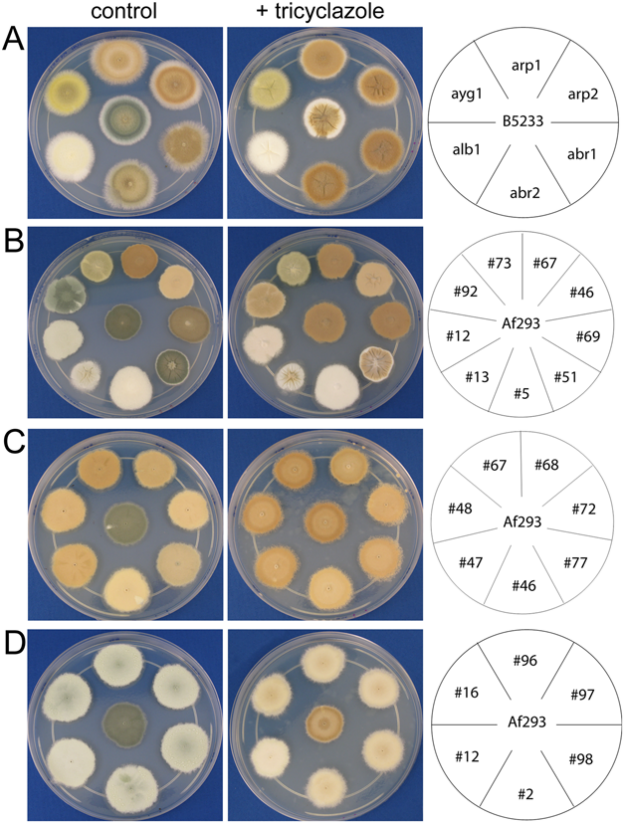
Tricyclazole sensitivity of selected *A. fumigatus* color mutants in the Af293 background and previously defined color mutants in the B5233 background. Images of *A. fumigatus* on YNB agar medium with and without tricyclazole after 3 days of growth at 30°C. (A) Wild type *A. fumigatus* strain B5233 and previously defined color mutants *alb1*, *ayg1*, *arp1*, *arp2*, *abr1*, and *abr2*; (B) wild type *A. fumigatus* strain Af293 and selected color mutant strains #5, #12, #13, #46, #51, #67, #69, #73, and #92 in the Af293 background; (C) brown color mutants #46, #47, #48, #67, #68, #72, and #77 in the Af293 background, and (D) light turquoise color mutants #2, #12, #16, #96, #97, and #98 in the Af293 background with and without tricyclazole present in agar medium.

To determine whether the enhanced pathogenicity of the color mutants towards *G. mellonella* was a strain-specific effect due to mutations in the B5233 genetic background or a general phenomenon caused by alterations in melanization, we decided to test the virulence of color mutants in the background of the sequenced strain Af293. Af293 has been shown to be genetically distinct from B5233 [54], [55]. These two strains are also morphologically distinct, with strain B5233 producing fluffier colonies (Figure 5A, B). If genetic background is responsible for the increased pathogenicity towards wax moth observed in the color mutants in the B5233 background, then a different virulence pattern would be expected for color mutants in the Af293 background. If the enhanced virulence to *G. mellonella* is caused by alteration of the melanization pathway, color mutants in the Af293 background would also exhibit higher pathogenicity level compared to the wild type strain. Furthermore, mutations in either other structural genes or regulators responsible for melanization that are not localized in the characterized gene cluster should also increase fungal virulence towards *G. mellonella*.

**Figure 6:**
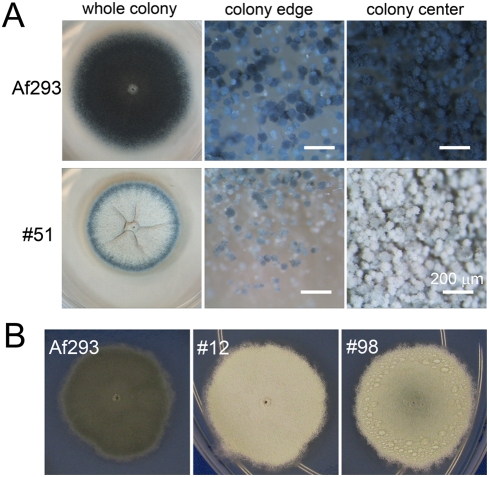
Colony morphology of novel color mutants. (A) Aged *A. fumigatus* mutant colony produces bluish grey conidia at the front edge and sand color conidia at the center of the mycelium. Strain #51 and wild type strain Af293 were incubated on YNB agar medium at 30°C for 1 week. From left to right are: images of the whole colony, conidiophores at the edge of the colony, and conidiophores in the center of the colony. Scale bars, 200 µm. (B) Light turquoise mutants, excluding #12, accumulate water droplets on the on the colony surface. The colony images of Af293, #12, and #98 were enlarged from Figure 5D.

To obtain conidiation color mutants in the strain Af293 background, we performed random insertional mutagenesis via *Agrobacterium* mediated transformation as described previously [56]. About 8000 hygromycin resistant insertional mutants were screened for alteration in conidial color. Over thirty mutants that displayed conidial colors different from the bluish-grey color of wild type were isolated. These mutants were grouped according to their conidial color. One mutant strain (underlined in the following strain lists) was chosen to represent each group and their colony morphology is shown in Figure 5B. Among these mutants, one (mutant #5) produced white conidia similar to the characterized *alb1* mutant; one (mutant #73) generated yellow conidia similar to the characterized *ayg1* mutant, and four (#15, #49, #69, and #71) generated different shades of brownish green color similar to characterized *abr2* mutant; eight (#46, #47, #48, #67, #68, #72, #77, and #116) produced different shades of brown color similar to the characterized *arp1* or *arp2* mutants (Figure 5A, B). In addition to the colors that have been observed previously, three novel conidial colors were also identified. One group of mutants (#34, #38, #39, #50, #51, #59, #79, #81, and #118) produced green conidia similar to wild type when the mycelium was young (Figure 5B). However, when the colony aged after long incubation (greater than 6 days), the conidia in the center of the colony turned light brown while the conidia at the front edge remain the wild type bluish-grey color, giving the colony a ring like look (Figure 6A). The second group of mutants (#2, #12, #16, #88, #92, #96, #97, and #98) produced conidia of different shades of light turquoise. All light turquoise mutants, excluding #12, tended to accumulate water droplets on the colony surface (Figure 6B). The third group of mutants (#13, #23, and #29) produced conidia of wheat color.

We tested the sensitivity of the selected color mutant representatives in the Af293 background and the previously defined color mutants (*alb1*, *arp2*, *arp1*, *abr1*, *abr2*, and *ayg1*) in B5233 background to the drug tricyclazole. Tricyclazole specifically inhibits reductases in the DHN-melanin biosynthesis pathway and causes accumulation of flaviolin, a shunt product of 1,3,6,8-THN. This observed response is similar to the response resulting from mutations in the reductase gene *arp2*, which also causes the formation of brownish color colony [48], [49], [57], [58]. As shown in Figure 5 (A, B), tricyclazole treatment caused both wild type strains B5233 and Af293 to turn brown. The defined *alb1* (white) and *ayg1* (yellow) mutants in B5233 background did not change color in the presence of tricyclazole, while all the other defined mutant colonies, including *abr1*, *abr2*, *arp1*, and *arp2*, turned brown, similar to the wild type strain B5233 (Figure 5A). This result is expected as the gene products encoded by *alb1* and *ayg1* act upstream of reductases in the melanin biosynthesis pathway [49]–[51]. Among the selected color mutants in the Af293 background, #5 (white) and #73 (yellow) did not change color in medium containing tricyclazole, while #13 (wheat), #12 (turquoise), #92 (dark turquoise), #69 (brownish green), and #51 (green color edge with sand color center) turned brown in the presence of tricyclazole. It is not certain if tricyclazole had any effect on strain #46 and #67 as they are lighter and darker brown even in the absence of tricyclazole (Figure 5B). Because several mutants producing different shades of the same color conidia, and only one representative of each color group was tested, it was not certain whether the observed phenotype in the presence of tricyclazole with the selected mutants would represent all of the other mutants that were placed into the same category. Therefore, all the mutants belonging to the turquoise and brown conidial color groups were further examined. The mutants with lighter shades of brown remained lighter in color in the presence of tricyclazole (Figure 5C) and all turquoise mutants changed to light brown with addition of tricyclazole in the medium (Figure 5D), similar to what was observed for the strains representing these groups. These results indicate that mutants grouped in the same color family behave similarly in the response to the inhibition of tricyclazole.

We further screened these color mutants for alterations (in-dels) in the six-gene cluster locus by PCR. Based on the analysis of PCR amplicons of the 19 kb locus (Figure S1), we detected mutations in the melanin biosynthesis gene cluster in several of the Af293 color mutants. For example, #5 is mutated in *alb1* gene, #48 is mutated in *arp1* gene, and #71 is mutated in *abr2* gene. These mutations and their conidial color phenotype are consistent. However, for the majority of the mutants, our screen did not detect any abnormality in the size of PCR amplicons of that locus. It is possible that these strains may harbor mutations (e.g. point mutations) in that locus that eluded our PCR screening, or alternatively but not mutually exclusively, some strains could have mutations in novel genes outside of the gene cluster that are also involved in the melanization. The latter hypothesis is highly possible given that strains producing novel conidial colors such as light turquoise were also isolated from this study (Figure 5), Although it is out of the scope of this current study, future investigation of these novel genes will likely reveal novel structural genes and regulators involved in the melanin biosynthesis in *A. fumigatus* and their characterization will certainly add more complexity to the understanding of the melanization pathway characterized thus far.

### 4. Conidiation color mutants in the Af293 background are highly pathogenic to *G. mellonella*

**Figure 7:**
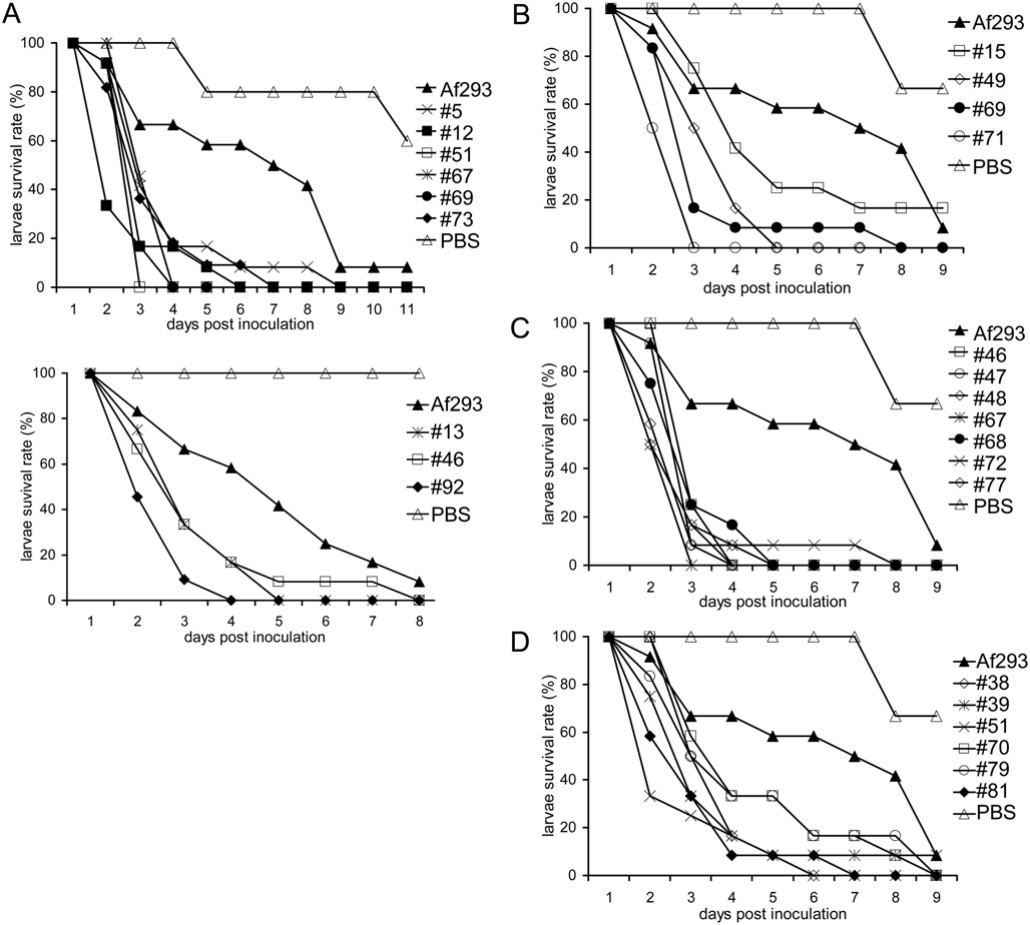
*A. fumigatus* color mutants in the Af293 background show enhanced virulence in the *G. mellonella* model. Larvae were inoculated with conidia of wild type and color mutants at concentrations of 1×10^6^ conidia per caterpillar. Percent *G. mellonella* survival was plotted against days post inoculation. (A) Color mutant representatives show an increased virulence that is statistically significant compared to the wild type strain Af293: #5 (p = 0.0193), #12 (p = 0.0038), #13 (p = 0.0062), #46 (p = 0.0067), #51 (p = 0.0038), #67 (p<0.0001), #69 (p<0.0001), #73 (p = 0.0003), and #92 (p = 0.0053). (B) Brownish green color mutants #49 (p<0.0001), #69 (p<0.0001) and #71 (p<0.0001) show increased virulence compared to the wild type strain Af293, while #15 (p = 0.1048) does not appear to have a statistically significant difference in virulence. (C) Brown color mutants all show increased virulence compared to the wild type strain Af293 that is statistically significant: #46 (p<0.0001), #47 (p<0.0001), #48, (p<0.0001), #67 (p<0.0001), #68 (p<0.0001), #72 (p<0.0001), and #77 (p<0.0001). (D) Color mutants, which produce green conidia at the front edge and brown conidia at the center of the colony as colonies age, show a statistically significant increase in virulence compared to the wild type strain Af293: #38 (p<0.0001), #39 (p<0.0001), #51 (p<0.0001), #70 (p = 0.0026), #79 (p = 0.0009), and #81 (p<0.0001).

**Figure 8:**
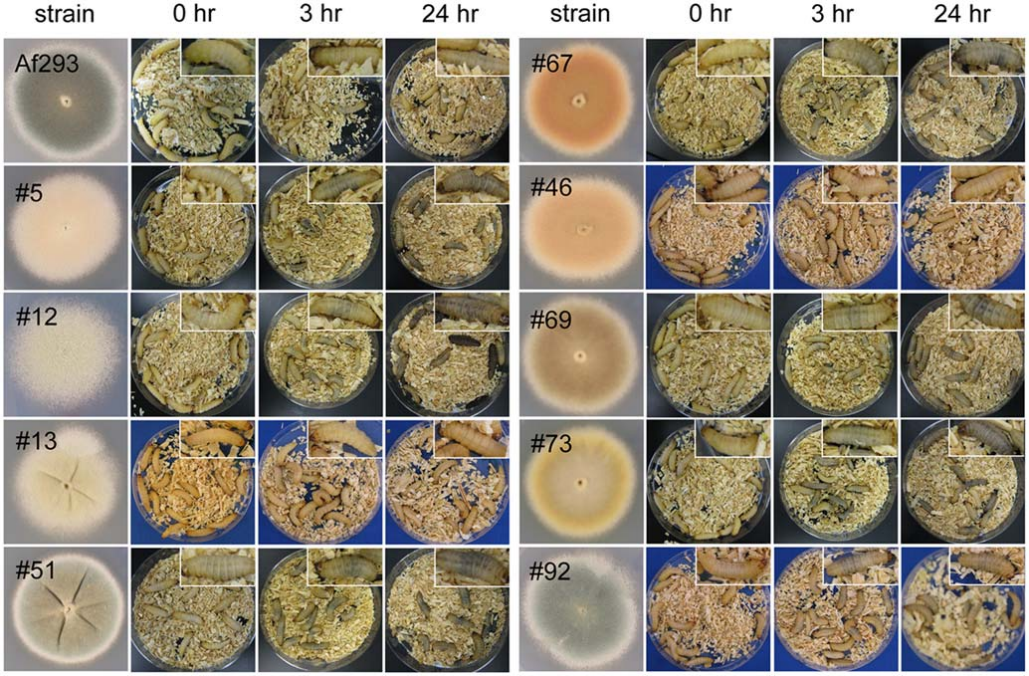
Infection with *A. fumigatus* color mutants in the Af293 background causes *G. mellonella* larvae to darken shortly after inoculation. Larvae were infected and *A. fumigatus* Af293 wild type strain and selected color mutants #5, #12, #13, #51, #67, #46, #69, #73, and #92 at the concentration of 1×10^6^ conidia per caterpillar. From left to right are images of *A. fumigatus* colonies, *G. mellonella* larvae at T = 0 h, 3 h, and 24 h post infection. Insets are close-up images of a representative single caterpillar from the same plate.

Upon isolation of conidiation color mutants in the Af293 background, we examined their pathogenicity in the wax moth larvae. One strain representing each color group was inoculated in the wax moth and the larvae survival rate was recorded as a function of time. As shown in Figure 7, the color mutants were more pathogenic to the insect than the wild type strain Af293. Similar to the situation with the color mutants in the B5233 background, the larvae darkened shortly after inoculation with spores of color mutants, and there again appeared to be a positive correlation between the darkening of the infected larvae and the pathogenicity of the strain (Figure 8). At least three independent experiments were performed for each strain and each showed a similar pattern. With the exception of white and yellow mutants, several strains producing varying shades of the same conidia color were isolated (for details, see result 3). To determine whether mutants producing varying shades of the same color family exhibit a similar trend in virulence, multiple members of several color groups were further examined for their virulence potential in wax moth. Seven strains producing brown conidia (#46, #47, #48, #67, #68, #72, and #77), four strains producing brownish green conidia (#15, #49, #69, and #71), and six strains producing ring-like colonies that are green at the edge and sand color in the center (#38, #39, #51, #70, #79, and #81) were used for wax moth virulence studies. As shown in Figure 7, all of the color mutants showed enhanced virulence albeit to different degrees. Our results support our hypothesis that alterations in melanin synthesis are mainly responsible for the enhanced virulence of *A. fumigatus* toward *G. mellonella*.

### 5. Conidial color mutants displayed varied sensitivity towards H_2_O_2_

Our virulence studies strongly support the idea that mutations in melanin biosynthesis pathway enhance *A. fumigatus* virulence potential towards wax moth. How these mutations increase virulence in wax moth is not yet clear. Because scavenging toxic oxygen radicals is a well-accepted function of melanin and the ability of melanin to quench reactive oxygen species is a major factor contributing to fungal resistance to the human immune system [46], [50], [59]–[61]. Therefore, we examined the sensitivity of color mutants toward oxidative stress to determine if this property correlated with their pathogenicity level in *G. mellonella*. All the representative color mutants showed a similar level of resistance towards superoxide stress based on similar growth in the presence of menadione bisulfate (data not shown). In contrast, the color mutants showed varied sensitivity towards H_2_O_2_, which is the key oxidizing agent and the source of other extremely toxic radicals produced by immunoactive cells to act against microorganisms.

**Figure 9:**
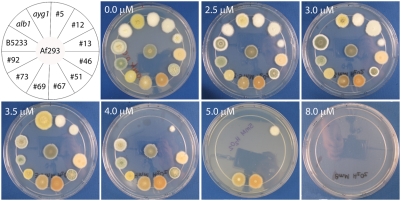
Color mutants display varied sensitivity towards hydrogen peroxide. *A. fumigatus* strains were cultured on YNB agar medium with 0 µM, 2.5 µM, 3.0 µM, 3.5 µM, 4.0 µM, 5.0 µM, and 8.0 µM H_2_O_2_ for 3 days at 30°C. Color mutants #12, #67, and #69 demonstrate an increased resistance to oxidative stress.

As shown in Figure 9, the *alb1* mutant in B5233 background was most sensitive to H_2_O_2_, followed by the *ayg1* mutant. This result is consistent with a previous study showing that a white color mutant of *A. fumigatus* obtained through UV mutagenesis is sensitive to hydrogen peroxide [62]. Color mutant #5 was one of the most sensitive strains in the Af293 background tested, which is consistent with our observations that #5 produced white conidia and bears a mutation in the *alb1* gene. Strain #13 (wheat color) was also highly sensitive towards H_2_O_2_, followed by #46 (light brown), #51 (green edge with sand color center), #73 (yellow), and #92 (dark turquoise). To our surprise, a few color mutants including #12 (light turquoise), #67 (brown), and #69 (brownish green) were more resistant to the H_2_O_2_ oxidative stress than the wild type strain Af293. Interestingly, these H_2_O_2_-resistant strains (#12, #67, and #69) were also among the most virulent strains to wax moth, as demonstrated earlier (Figure 7). Although sensitivity towards H_2_O_2_ oxidative stress alone does not appear to be a good indication of pathogenicity level in *G. mellonella*, it is likely that resistance to H_2_O_2_ further increases *A. fumigatus* virulence potential.

### 6. Color mutants have the same germination rate as their parental wild type strains

Under the conditions used in this study, all the *A. fumigatus* strains were able to kill *G. mellonella* larvae. Based on the observations that free *A. fumigatus* hyphae have been occasionally observed in the extracted *G. mellonella* hemolymph after 24 hours post inoculation (Figure S2), conidia must be able to germinate and grow as hyphae inside the *G. mellonella* larvae. During germination, resting conidia of *A. fumigatus* break dormancy and grow isotropically. The swollen conidia then start to become pear shaped and send out germ tubes. The germ tubes then elongate to become true hyphae (Figure S3)[63]. It is reported that *G. mellonella* hemocytes are inefficient in phagocytosing *A. fumigatus* germinated conidia *in vitro* and pre-germinated conidia are highly virulent to caterpillar *in vivo* [21]. Consequently, a faster germination rate could significantly enhance fungal virulence in this insect model. Therefore, we examined the rate of germination in representative color mutants and compared it to that of wild type. Conidia of *A. fumigatus* strains (Af293, #5, #12, #67, #69, B5233, and *alb1*) were cultured in YPD media at 37°C with shaking. We measured the conidial size of 100 conidia at each time point (0, 3, 5, and 7 hours) during germination and we did not detect any significant difference among these different strains (Figure S3). These results exclude the possibility that faster germination rates are responsible for the higher virulence potential of color mutants.

### 7. Activation of *G. mellonella* innate immune responses prior to fungal infections narrows the differences in virulence between *A. fumigatus* wild type strains and color mutants

To interpret the seemingly contradicting findings that *G. mellonella* larvae mounted drastic immune responses against *A. fumigatus* color mutants as evidenced by the rapid melanotic capsule formation, and that the color-mutant-infected larvae had higher mortality rates, we hypothesize that over-reactive immune responses induced by color mutants may become harmful to the insect, which may have compromised its ability to clear live fungal infections.

**Figure 10:**
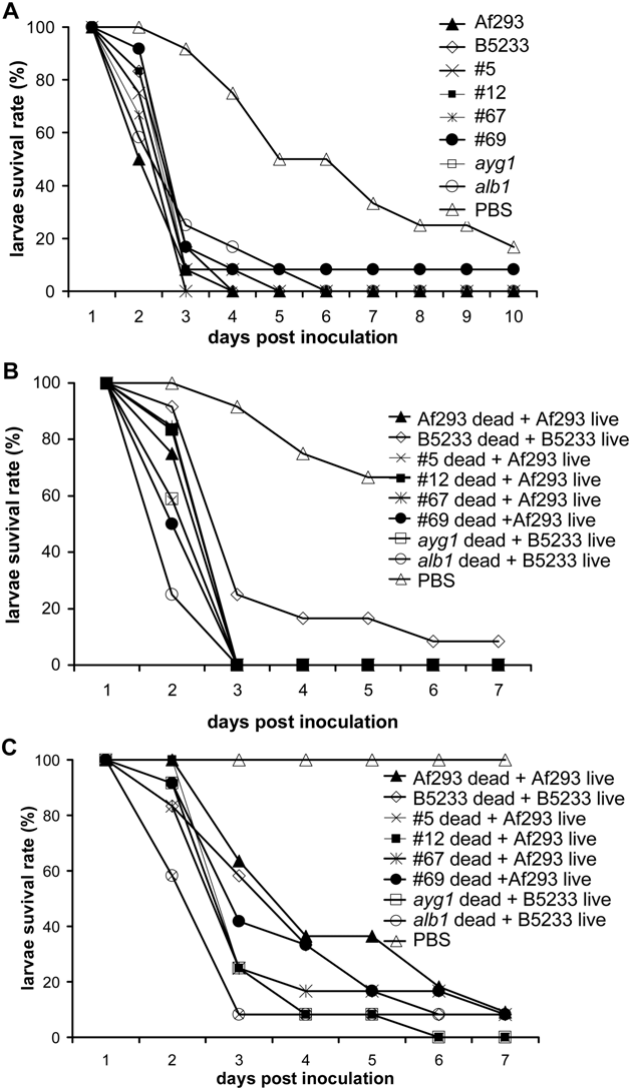
Activation of insect immune responses enhances virulence of wild type strains. (A) Activation of insect immune responses prior to fungal infection narrows the virulence differences between the color mutants and the wild type strains. Larvae were incubated at 37°C for 24 hours before inoculation with Af293, B5233, #5, #12, #67, #69, *ayg1*, and *alb1*. The p values when compared to the corresponding wild types strains are listed as follows: #5 (0.2213), #12 (0.0061), #67 (0.676), #69 (0.0136), *ayg1* (0.3409), and *alb1* (0.8099). (B–C) Mixed infections with dead spores of color mutants and live spores of wild type strains increase larva mortality. (B) Larvae were inoculated with 10 µl of indicated spore mixtures (1×10^6^ dead conidia and 1×10^6^ live conidia per insect). (C) Larvae were inoculated with 5 µl of indicated spore mixtures (5×10^5^ dead conidia and 5×10^5^ live conidia per insect). The *p* values when compared to the mixture of corresponding dead and live wild type spores are listed as follows : #5 dead+Af293 live *vs* Af293 dead+Af293 live (0.3297); #12 dead+Af293 live *vs* Af293 dead+Af293 live (0.7913); #67dead+Af293 live *vs* Af293 dead+Af293 live (0.9436); #69 dead+Af293 live *vs* Af293 dead+Af293 live (0.7212); *alb1* dead+B5233 live *vs* B5233 dead+B5233 live (0.0734); and *ayg1* dead+B5233 live *vs* B5233 dead+B5233 live (0.093).

We speculate, based on the hypothesis that activation of the insect immune responses prior to challenge with color mutant or wild type fungal strains would only decrease the survival of infected wax moth larvae and diminish the differences in the pathogenicity levels observed between color mutant and wild type strains. Indeed, incubating the larvae at 37°C for 24 hours prior to fungal inoculation, a treatment previously shown to activate the immune responses in *G. mellonella* larvae [64], reduced the preceived differences in virulence between color mutants and the wild type controls (Figure 10A).

### 8. Co-inoculation of live wild type spores with dead spores of color mutants increase larva mortality rates

Although the larvae darkening might compromise the ability of the insect to clear live fungal infections, this does not affect larva viability. This is based on our observations that dead spores of *A. fumigatus* color mutants in both B5233 and Af293 backgrounds induced similar levels of larvae darkening but failed to kill the insect (data not shown). This result also suggests that alterations in conidial surface of the color mutants might be a major factor responsible for the larvae darkening. If over-reactive immune responses can be induced by dead color mutants, we speculate that the larvae will show increased mortality rates when infected with live spores of wild type strains with dead spores of color mutants.

So we examined the larvae inoculated with mixtures of dead conidia of color mutants and live wild-type conidia. Initially, we inoculated the larvae with 1×10^6^ dead color mutant conidia and 1×10^6^ live wild type conidia per insect, as the dose of 1×10^6^ live conidia per larva has been used in all other virulence experiments. The infected larvae of all treatment groups turned dark soon after inoculation. All infected larvae died within three days post inoculation, except the control group infected with wild type B5233 dead spores mixed with wild type B5233 live spores (Figure 10B). This pattern is similar to what we observed in larvae that were infected with high doses of live wild-type conidia (Figue 1 with 1×10^7^ conidia per larva) or in the larvae whose immunity was pre-activated (Figure 10A). This again supports our hypothesis that over-charged immunity may be responsible for the increases larva mortality when challenged with live fungal infections.

Because it is difficult to detect any differences in virulence between groups due to the rapid death of the infected larvae at that dose, we reduced the dose by half and inoculated the larvae with 5×10^5^ dead and 5×10^5^ live conidia per insect. As shown in Figure 10C, differences between groups can be detected with this change although the statistical significance for the pattern shift is relatively low. The larvae infected with a mixture of dead color mutant conidia and live wild-type conidia showed a trend with increased mortality rates compared to those infected with a mixture of dead and live spores of only wild-type strains (Figure 10C). This again suggests that excessive immune responses induced by dead spores of color mutants may enhance the larva killing effect of wild type *A. fumigatus* strains. These observations also suggest that toxic compounds secreted by live color mutants during infection are not likely to be a major factor responsible for the enhanced virulence of color mutants.

## Discussion

Because melanin is a mixture of negatively charged hydrophobic macromolecules and is abundant on the conidial surface, mutations in melanin biosynthesis could lead to significant alterations in the morphology and cell surface properties. For example, *A. fumigatus* wild type conidia have an echinulate surface while white, *alb1* color mutants produce smooth surface conidia [46], [62]. As demonstrated in *C. neoformans*, melanized cells are also less porous than non-melanized cells [65]. Absence of melanin in *A. fumigatus* could give larger molecules access to the inner cell wall, which is otherwise restricted, and lead to exposure of cell wall components including the pathogen-associated molecular patterns (PAMPs) that are normally masked by melanin. For example, deletions of *alb1* and *arp1* significantly increase the binding of the human complement component C3 to *A. fumigatus* conidia [46], [51]. Additionally, disruption of the *alb1* gene results in enhanced exposure of β-(1,3) glucan, a polysaccharide in the fungal cell wall that is a major target of the mammalian innate immune system [66]. Such changes could lead to enhanced immune responses from the mammalian host. Indeed, the *alb1* conidia stimulate neutrophils to release more reactive oxygen species than wild-type conidia [62] and these mutant conidia also undergo phagocytosis [67] and traffic to phagolysosomes more readily [68]. During *A. fumigatus* infection in mammals, white color mutants likely encounter more robust inflammatory responses from the host, which may account for the decrease in virulence of these mutant strains in animals [46], [62], [68]. As β-(1,3) glucan in the fungal cell wall is also a primary inducer of insect innate immune system [26], [69], *A. fumigatus* color mutants might trigger an excessive immune response in the wax moth due to increased exposure of β-(1,3) glucan on the cell surface. The induction of insect darkening by dead conidia of *A. fumigatus* color mutants and the inability of the dead spores to cause mortality in the wax moth again support our hypothesis that the altered conidial surface of color mutants may trigger the strong reaction of the wax moth immune system.

Alternatively, but not exclusively, defects in melanin production in the cell wall of the *A. fumigatus* color mutants could also allow more efficient release of fungal proteolytic enzymes, which may also induce excessive immune responses within the host. It is known that metalloproteinases from pathogens represent potent elicitors of innate immune responses including melanization in *G. mellonella* [70]–[72]. Increased metalloproteinase activity in color mutants may contribute to the augmented ability of color mutants to induce aggravated immune responses from infected larvae. However, upon assaying for general metalloproteinase activity in metabolically active conidia, we failed to elucidate a relationship between increased mortality and metalloproteinase activity in the color mutants when compared to the observed metalloproteinase activity of the wild type strains (Table S1).

Although fungal melanin has been shown to be important for virulence in mammal models for several pathogenic fungi, including *A. fumigatus*, results from the current study demonstrate instead, that lack of melanin enhances *A. fumigatus* virulence in the insect *G. mellonella*. The difference in the observed pathogenicity of conidiation color mutants in these different host models is most likely attributed to the difference in host immune responses induced by the color mutants. In mice, color mutants *alb1* and *arp1* induce a more robust immune response, resulting in reduced virulence of these mutants. Here, we hypothesize that the exacerbated immune response of the wax moth larvae to *A. fumigatus* color mutants is one of the major factors responsible for the morbidity of these larvae, possibly triggered by the altered surface properties of the color mutant conidia. Consequently, the over-reactive and excessive immune responses from these infected larvae might impede the insect's ability for subsequent clearing of live fungal infections.

Our hypothesis is plausible based on previous studies in other insects showing that immune responses bear costs [73], [74] and can cause self-damage [75]. For example, the phenoloxidase cascade that catalyses the production of melanin in insects also generates phenols, quinones, and other highly cytotoxic molecules [76]–[78]. Although the process of melanoic capsules formation is a key defense mechanism against a wide range of pathogens mediated by the insect prophenoloxidase system [26], [79], [80], melanization of self-tissue is also one of the phenotypic consequences of phenoloxidase-derived self-harm and reduced tissue functions as demonstrated in the mealworm beetle *Tenebrio molitor* [75].

Our hypothesis that excessive immune responses induced by color mutants might impede the insect's ability for subsequent clearing of live fungal infections is also supported by our seemly contradictory observations that dramatic melanoic capsules rapidly formed upon the inoculation of color mutants, an indication of rapid and strong activation of host defense responses, while color-mutant-infected larvae showed increased mortality rates. Our observations that co-inoculation with dead spores of color mutants enhanced the killing effect of the live wild type spores, and that activation of insect immune system prior to fungal infections increase larva mortality and narrowed the virulence differences observed between the color mutants and the wild type strains support the idea that altered conidial surface of color mutants triggers the over-reaction of the wax moth immune system, which impairs the ability of the insect to combat live fungal infections, leading to increased mortality rates in infected larvae. This phenomenon is similar to the immunopathology in mammalian systems, where diseases are caused by oversensitive host immune responses. Although it is possible that toxic byproducts accumulated by color mutants could contribute to the increased larva mortality rates given that several byproducts of the DHN-melanin pathway have been shown to have antibacterial or immunosuppressive properties [81], their contribution, if exist, would be minor given that dead spores of color mutants enhanced the virulence of the live wild type spores during co-inoculation. Furthermore, no apparent alterations in secondary metabolites such as gliotoxin were detected in color mutants compared to wild type strains (data not shown). Taken together, our observations suggest that toxic byproducts accumulated by color mutants are not likely to be responsible for the increased larva mortality rates at any significant level.

Subsequently, it would be interesting to study melanin defective mutants in native insect fungal pathogens that are currently being used or have the potential to be used as biological insecticides. If melanization defective strains of these fungi display increased virulence against insects through natural routes of infection (cuticle exposure or ingestion) and cause no harm to mammals, these mutants will not only be more effective insecticides, but also have less environmental impact in the field. In addition, non-melanized mutants are more sensitive to radiation and other treatments, which could provide additional/complementary means to eradiate these fungal strains when necessary.

Regardless of the host system used, it is conceivable that the net effect of multiple fungal factors (positive and negative) acting together influence the final outcome of the infection. Therefore, it is not surprising that environmental opportunistic human pathogens like *A. fumigatus* deploy overlapping, but distinct, strategies to ensure fungal survival in different host systems. Thus phenotypic differences in traits such as susceptibility to oxidative stress, germination rate, hyphal growth, and production of secondary metabolites such as mycotoxins influence the outcome of infection. The observation that color mutant strains that are resistant to concentrations of hydrogen peroxide greater than 5 µM are also highly virulent in the insect *G. mellonella*, suggests that fungal resistance to oxidative stress is one of the key factors necessary for the infection of the caterpillar as well as mammals. Although we did not detect any differences in growth or gliotoxin production in these mutants, other unknown phenotypical characters of these color mutants could play a role during infection.

Our study also revealed additional limitations of using the *G. mellonella* model for inferring the pathogenic potential of *A. fumigatus* strains in mammals. Some virulence traits, such as melanin, that are important for mammalian infection may not necessarily be required for *G. mellonella* infection and *vice versa*. For example, the pathogenicity level of *Aspergillus* species in mammals does not correlate with the species' pathogenicity level in the wax moth. Of the species in the genus *Aspergillus*, *A. fumigatus* is the most virulent in mammals, but *A. flavus* isolates are much more virulent to *G. mellonella* compared to *A. fumigatus* isolates [47]. Similar phenomenon might also exist for different species of *Cryptococcus* [13].

In this study, we found that the dose of 1×10^6^ resting conidia per *G. mellonella* larva is suitable for virulence studies of *A. fumigatus* strains B5233 and Af293. This inoculum is more or less in line with the inoculum used for studying other pathogenic yeasts [23], [82]. However, our finding is different from a previous report showing that dormant/resting conidia are avirulent unless the inoculation density of 1×10^7^ conidia per insect is used [21]. The observed difference could be caused by a difference in *A. fumigatus* genetic background, as strain ATCC 26933 was used in the previous study. Different incubation temperatures for infected larvae may also contribute to the observed differences in pathogenicity. The previous study incubated infected larvae at 30°C, while 37°C, the more common mammalian body temperature was used in the current study. Temperature has been shown to be an important factor regulating immune responses in *G. mellonella* [64], and higher temperature used in this study might render the larvae more susceptible to fungal infections.

Our current data show that larvae of *G. mellonella* could provide a reproducible model for *A. fumigatus* studies, providing that the virulence traits are required for fungal infections in both mammals and the insect. It also underscores the importance of understanding the innate immunity of the insect host in providing insights into the mechanisms underlying the difference in the pathogenicity level of different fungal strains in these insect models. The possibility of using insect models as compared to mammal animal models for *in vivo* pathogenicity testing and screening offers a lot of advantages, which necessitate further investigation of this insect model as an additional valuable tool to study this important fungal pathogen.

## Materials and Methods

### Strains and growth conditions

*A. fumigatus* strains B5233, *alb1*, *arp1*, *arp2*, *abr1*, *abr2*, and *ayg1* were kindly provided by Dr. June Kwon-Chung at NIH. Strain Af293 was provided by Robert Cramer Jr. at Montana State University. All the color mutants in the strain Af293 background were generated by random insertional mutagenesis via *Agrobacterium* mediated transformation for this study. Strains were grown on Yeast Nitrogen Base medium (YNB) with 2% agar at 30°C for 4 days unless specified otherwise. Conidia produced after 4 days of incubation were collected with PBS buffer supplemented with 0.1% (v/v) Tween-80 surfactant and filtered through four layers of miracloth. Cell density was determined by counting using a hemacytometer.

### Inoculation of G. mellonella larvae

Wax-moth (about 0.3–0.4 gram in body weight) in the final instar larval stage (Vanderhorst, Inc., St. Marys, Ohio) was used (12 per strain). Larvae were stored in wood shavings in the dark at 22°C prior to use. Infection of *G. mellonella* larvae was essentially the same as previously used for *C. neoformans* [13]. Each wax-moth larva was infected with 1×10^6^ (or concentrations as indicated) *A. fumigatus* conidia in 5 µl PBS by injection into the hemocoel of each wax-moth via the last left proleg. After injection, the wax-moth larvae were incubated in plastic containers at 37°C in a moist chamber and monitored daily. Caterpillars showing signs of severe morbidity, such as no response to touch and change of body color, were sacrificed by cold treatment at −20°C. The survival rate of wax-moth was plotted against time, and *p* values were calculated using the student *t* test. At least three experiments were performed for each strain and only one experiment result is shown.

### Infection of G. mellonella larvae with mixtures of live and dead spores

Spores were boiled for 20 minutes at 100°C. The death of the boiled conidia after this treatment was confirmed by plating. The boiled spores were washed three times with PBS buffer, and then suspened to the final concentration of 2×10^8^ condia/ml. Equal volumes of dead spores were mixed with live spores of indicated strains at the same concentration. 5 µl or 10 µl of the mixture (5×10^5^ or 1×10^6^ live conidia per insect) was used for larva infection.

### Random insertional mutagenesis via Agrobacterium mediated transformation (AMT)

Insertional mutagenesis via AMT was performed essentially as previously described with modifications [56]. Basically, the Ti plasmid pBHt2 that contains a hygromycin resistance marker [83] was transformed into the *Agrobacterium* strain EHA105. The resulting *Agrobacterium* cells were cocultured with *A. fumigatus* on induction medium supplemented with 100 µM of acetosyringone, which stimulates *Agrobacterium* to transfer the T-DNA into the fungal genome. After three day coincubation, *A. fumigatus* transformants were selected on *Aspergillus* minimal medium supplemented with hygromycin (200 µg/ml) and cefotaxime (100 µg/ml). The antibiotic cefotaxmine cleared *Agrobacterium* cells.

### Screening for conidiation color mutants

All the hygromycin resistant *A. fumigatus* transformants were grown on YNB agar medium with hygromycin (200 µg/ml) at 30°C for 4 days. Strains that displayed different conidial color from the wild type bluish-grey were selected and streaked for pure culture.

### Tricyclazole sensitivity test

Sensitivity assay towards tricyclazole was essentially performed as described previously [46]. Tricyclazole was dissolved in ethanol to make 3 mg/ml stock and was added to YNB agar medium to make final concentration of 30 µg/ml. YNB medium with 1% EtOH alone or no EtOH were used for comparison to exclude the possibility that 1% EtOH might affect morphology or conidial pigmentation. Conidia of *A. fumigatus* strains were inoculated on the plates and incubated at 30°C for 4 days.

### Oxidative stress assay

Approximately 1 µl conidial suspension (2×10^8^ conidia/ml) of each strain were spotted onto YNB agar with the addition of 0 µM, 0.1 µM, 0.5 µM, 2 µM, 10 µM, 25 µM, 50 µM, 100 µM menadione bisulfate and allowed to grow at 30°C for 3 days. Similarly, 1 µl of 2×10^8^ conidia of each strain were spotted onto YNB agar with the addition of 0 µM, 5 µM, 20 µM, 100 µM, 500 µM, 1 mM, and 5 mM H_2_O_2_ and allowed to grow at 30°C for 3 days. After the initial data was gathered, this experiment was repeated and modified by inoculating 1 µl conidial suspension (2×10^8^ conidia/ml) of each strain onto YNB agar containing concentrations of 1 mM, 1.5 mM, 2 mM, 2.5 mM, 3 mM, 3.5 mM, 4 mM, 4.5 mM, 5 mM, 6 mM, 7 mM, 8 mM H_2_O_2_ and allowed to grow at 30°C for 3 days.

### Germination assay

Conidia of mutants (#5, #12, #67, and *abl1*) and of their corresponding wild type strains (Af293 and B5233) were inoculated in YPD medium (1% yeast extract, 2% BactoPeptone, and 2% dextrose) to make the final cell concentration 3×10^6^ conidia per ml, which was roughly equivalent to the dose used in wax moth studies. Strains were cultured at 37°C for 0 hour, 3 hours, 5 hours, and 7 hours with shaking. At indicated time point, cells were fixed with 3.7% formaldehyde in PBS. Cell size was determined by measuring the cell diameter from digital images captured with a camera connected to a light microscope. 100 randomly chosen cells were examined for each strain at each time point and only resting conidia and swollen conidia were measured for conidial size based on photographs. Images of a scale slide taken at the same microscope condition were used for scale calculation.

### Genomic DNA purification

Strains were inoculated in 25 ml YPD liquid medium supplemented with hygromycin (200 µg/ml) and incubated at 30°C with shaking for three days. The hyphae pellet was collected by centrifugation and aspiration. The cell pellet was frozen immediately at −80°C, lyophilized overnight, and stored at −20°C until genomic DNA was prepared using the CTAB protocol as described previously [84].

### Screening alterations in the melanin biosynthesis six-gene cluster by PCR and sequencing

Alteration of selected color mutants in the six-gene cluster was screened by PCR using primers that covered the whole region. Primers used are listed below and primer locations in the gene cluster are shown in Figure S1. Selected mutants that have alterations in that region detected by this PCR method were further confirmed by sequencing. Linlab 41 (GTCTCCCAGACCAAGGCC), Linlab 42 (GCGCTCGGCTTGCTTC), Linlab 43 (CCCTTTTCAATGATCTCCG), Linlab 44 (GGTGTGCTGCGGGCG), Linlab 45 (GACCAGCGACATCGCC), Linlab 46 (GCGTAGGTGTTGGCCG), Linlab 47 (CCCTGGAGTCCATCGAAC), Linlab 48 (CCACGACGGCTCCATC), Linlab 49 (GGCTGGCTGTCGTGTCG), Linlab 50 (GACGGCCATGTAACACCC), Linlab 51 (CAGGTGTTCACCATTCCG), Linlab 52 (TCCGCTCTGGGAGATCAG), Linlab 53 (GGATGACGGGCGTCG), Linlab 54 (CAGGCGTGAATGCTCGG), Linlab 55 (GACCGCGTCGGCATC), Linlab 56 (CGCTGTAGTTCGACTCCG), Linlab 57 (CCACGAACCTGCCGTCC), Linlab 58 (CCTGCCGGTCACCGTC), Linlab 59 (CAACTTCGCCGACTACGG), Linlab 60 (GGCGCCTGAGGCTGC), Linlab 61 (CCTCGATCATTGTGGACG), Linlab 62 (GCGTCAGTGGGCAAAGG), Linlab 63 (ACCCACGTATCGTTACGG), Linlab 64 (CGGACGCGCTCAAGATC), Linlab 65 (GCTTCCTTGGCCCGG), Linlab 66 (CCCGTGCTTGGTTGCC), Linlab 67 (GGAGGCGCAAACATCTG), Linlab 68 (ACTGGGCGAGACAATTCC), Linlab 69 (CACGCCGTCGACCTTG), Linlab 70 (CACGGACAGCACCTTGC), Linlab 71 (GGTGGTGCACTGATGGTG), Linlab 72 (CCTCGTCGCAACCGTAC), Linlab 73 (CCACTGCGGTGACCCTG), Linlab 74 (GCCGGCGAATGAGCG), Linlab 75 (CACCATTCCTCGCTGCA), Linlab 76 (CTGCAGGACAAGGCGCA), Linlab 77 (GCGAGTCGAGCAGCAGC).

## Supporting Information

Figure S1 Locations of primers used for PCR screening within the six-gene cluster for melanin biosynthesis.
(0.08 MB DOC)

Figure S2 *A. fumigatus* hyphae isolated from *G. mellonella* haemolymph. Larvae were infected with *A. fumigatus* B5233 wild type strain. Haemolymph was collected after 24 hours post inoculation and was immediately examined microscopically. Scale bar, 10 µm.
(2.22 MB TIF)

Figure S3 Size distribution of color mutants and their corresponding wild types at different stages of the germination process. Conidia were cultured in YPD media at 37°C with shaking for the indicated time, fixed, and then photographed. Cell size was determined by measuring the cell diameter from digital images. The image on the top shows the germination process: resting/dormant conidia (a), swollen conidia (b, c), pear shaped conidia (d), germ tubes (e), and hyphae (f). The bottom graph shows the conidia size distribution of each strain at 0, 3, 5, and 7 hours.
(1.00 MB TIF)

Table S1 Metalloproteinase activity in color mutant and wild type strains
(0.03 MB DOC)
