# Supplementary figures and images for: Conidiation Color Mutants of Aspergillus fumigatus Are Highly Pathogenic to the Heterologous Insect Host Galleria mellonella
Source: PLoS One. 2009 Jan 19;4(1):e4224. doi: 10.1371/journal.pone.0004224 (PMC2625396; doi:10.1371/journal.pone.0004224)

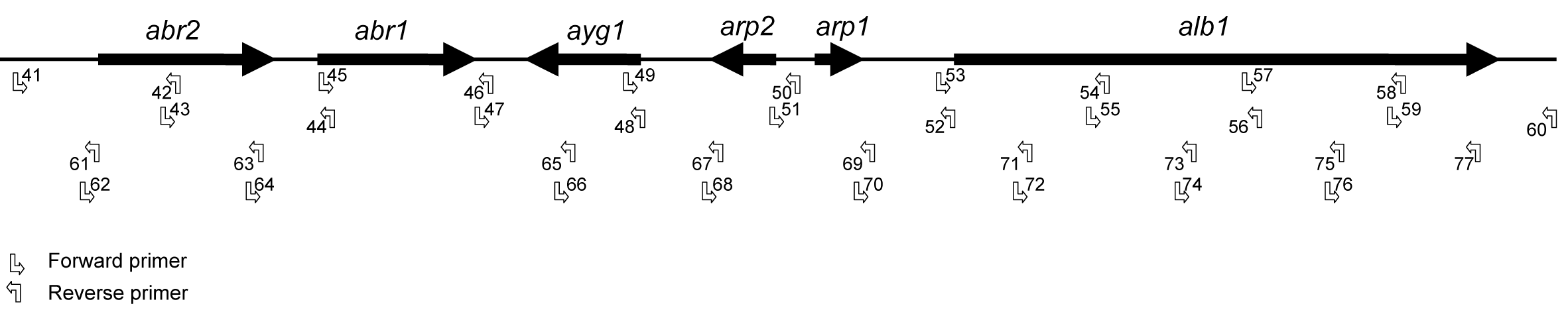

Supplement: Figure S1 — Locations of primers used for PCR screening within the six-gene cluster for melanin biosynthesis. (0.08 MB DOC) [file pone.0004224.s001.doc]

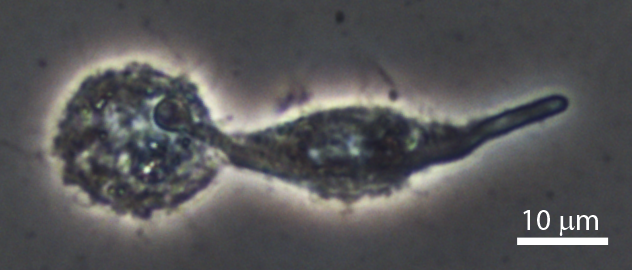

Supplement: Figure S2 — A. fumigatus hyphae isolated from G. mellonella haemolymph. Larvae were infected with A. fumigatus B5233 wild type strain. Haemolymph was collected after 24 hours post inoculation and was immediately examined microscopically. Scale bar, 10 µm. (2.22 MB TIF) [file pone.0004224.s002.tif]

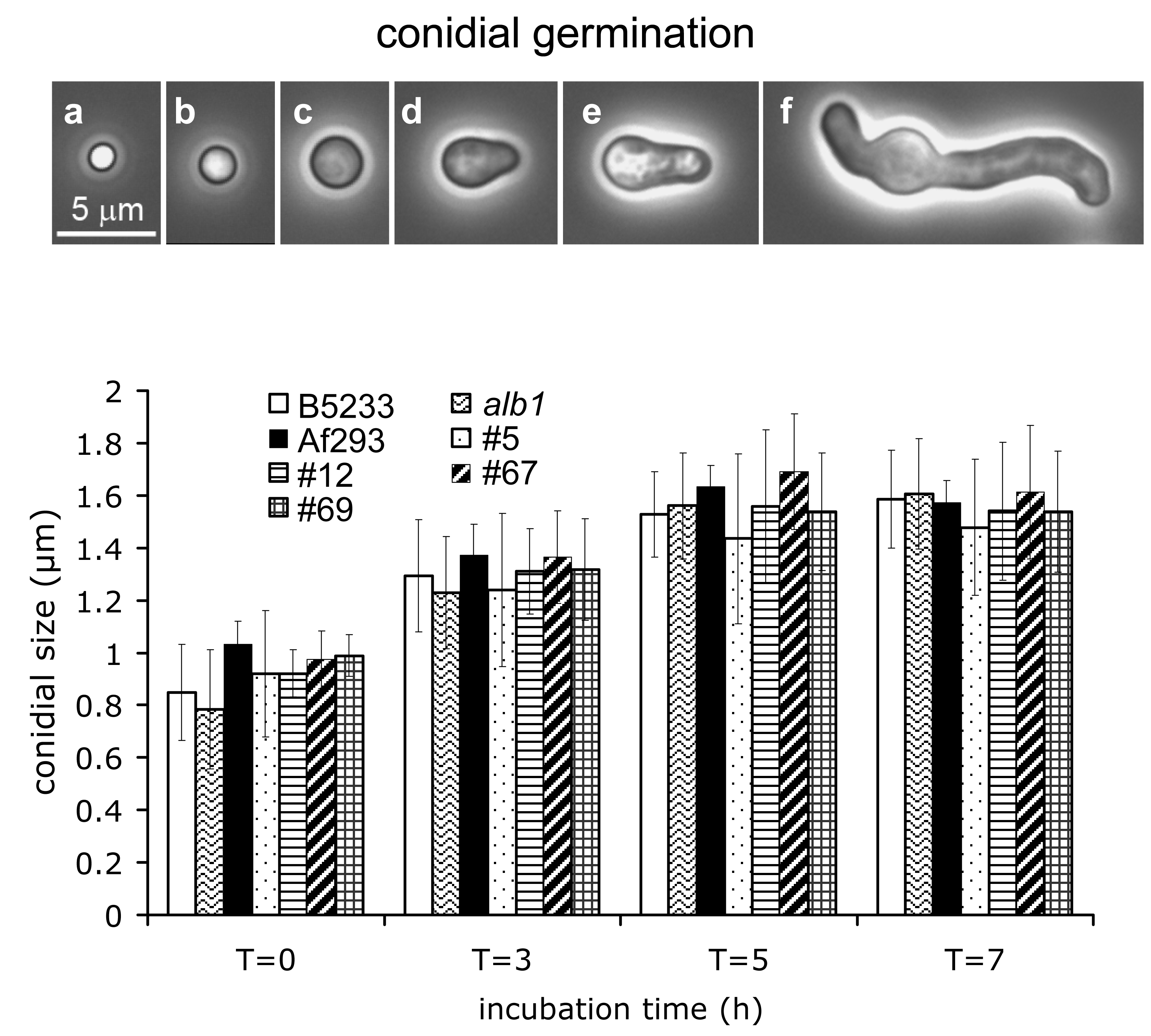

Supplement: Figure S3 — Size distribution of color mutants and their corresponding wild types at different stages of the germination process. Conidia were cultured in YPD media at 37°C with shaking for the indicated time, fixed, and then photographed. Cell size was determined by measuring the cell diameter from digital images. The image on the top shows the germination process: resting/dormant conidia (a), swollen conidia (b, c), pear shaped conidia (d), germ tubes (e), and hyphae (f). The bottom graph shows the conidia size distribution of each strain at 0, 3, 5, and 7 hours. (1.00 MB TIF) [file pone.0004224.s003.tif]
