# Supplementary material for: Conidiation Color Mutants of Aspergillus fumigatus Are Highly Pathogenic to the Heterologous Insect Host Galleria mellonella
Source: PLoS One. 2009 Jan 19;4(1):e4224. doi: 10.1371/journal.pone.0004224 (PMC2625396; doi:10.1371/journal.pone.0004224)
Supplement: Table S1 — Metalloproteinase activity in color mutant and wild type strains (0.03 MB DOC) [file pone.0004224.s004.doc]

Supplemental Table 1: Metalloproteinase activity in color mutant and wild type strains

| *A. fumigatus* Strain | Maximum Fluorescence Signal (AU) |
| --- | --- |
| B5233 | 6710 ± 290 |
| *alb1* | 8340 ± 180 |
| *ayg1* | 7420 ± 360 |
| Af293 | 9910 ± 330 |
| #5 | 9770 ± 320 |
| #12 | 10100 ± 190 |
| #67 | 15200 ± 510 |
| #69 | 6630 ± 270 |

**Supplemental Methods:**

*Metalloproteinase activity assay in wild type and mutant A. fumigatus strains.* To assay potential differences in levels of metalloproteinase activity between wild type and mutant *A. fumigatus* strains EnzChek Gelatinase/Collagenase Assay Kit (Molecular Probes E-12055) was used. This kit utilizes a gelatin-fluorescein conjugate (D-12054) as the proteolytic substrate. Wild type and mutant *A. fumigatus* strains were normalized to concentrations of 3x10^7^ spores/ml in YNB media and incubated for 3 hours at 37ºC. Approximately 3x10^6^ germinated spores/ml were assayed in 200 µl of assay mixture containing 1 mg/ml of DQ gelatin-fluorescein conjugate in a reaction buffer of 50 mM Tris-HCl pH 7.6, 150 mM NaCl, 5 mM CaCl2, 0.2 mM sodium azide at 37º C. The increase in fluorescence emission intensity as a function of time was measured at 495/515 nm at five-minute intervals using a 96-well fluorescent plate reader. Fluorescence emission intensity was corrected for background fluorescence and maximum fluorescence emission intensity was determined graphically using KaleidaGraph (Synergy Software).
